# Development of a system for efficient callus production, somatic embryogenesis and gene editing using CRISPR/Cas9 in Saffron (*Crocus sativus* L.)

**DOI:** 10.1186/s13007-020-00589-2

**Published:** 2020-04-07

**Authors:** Sudha Chib, Arulprakash Thangaraj, Sanjana Kaul, Manoj Kumar Dhar, Tanushri Kaul

**Affiliations:** 1 Genome Research Laboratory, School of Biotechnology, University of Jammu, Jammu, Jammu and Kashmir India; 2 Nutritional Improvement of Crops Group, Department of Plant Biology and Biotechnology, International Centre for Genetic Engineering and Biotechnology (ICGEB), New Delhi, India

**Keywords:** Cas9, CRISPR, Gene manipulation, Saffron, Invitro regeneration, Transformation

## Abstract

**Background:**

*Crocus sativus* is a recalcitrant plant for genetic transformation and genetic improvement, largely due to difficulties in *Agrobacterium* mediated transformation and vegetative reproduction. Effective genome editing requires proficient callus production and an efficient method to deliver Cas9 and sgRNAs into the plant. Here, we demonstrate *Agrobacterium*-mediated transformation of saffron. Further, we developed a CRISPR-Cas9 based system in this plant, for efficient gene knockout or edits in future.

**Results:**

Efficient callus production and regeneration confers important benefits in developing competent transformation system in plants. More than 70% multiplication rate of callus initiation was achieved from corm slices of saffron subjected to a two-step sterilization procedure and grown on complete MS medium supplemented with 2,4-D (0.5 mg/L), BAP (1 mg/L), IAA (1 mg/L), photoperiod of 16/8 h and 45% relative humidity at 20 ± 2 °C. In vitro cormlet generation was accomplished in 8 weeks by using mature somatic embryos on MS medium supplemented with TDZ (0.5 mg/L) + IAA (1 mg/L) + Activated charcoal (0.1 g/L) at 15 ± 2 °C. The attempt of using *Agrobacterium*-mediated transformation resulted in successful integration of the binary vector into the somatic embryos of saffron with a transformation efficiency of 4%. PCR and Southern blot analysis confirmed the integration of Cas9 into saffron.

**Conclusion:**

The protocol for callus production, somatic embryogenesis and regeneration was standardised. Successful demonstration of integrated Cas9 in this study constitutes first step in developing strategies for genetic manipulation of saffron, which has so far been considered recalcitrant. Furthering the development of this technology holds significant potential for advancing genetic research in saffron by integrating multigene targeting and/or use of recyclable cassettes.

## Background

The dried stigmas of *Crocus sativus* constitute saffron of commerce, which is considered to be the costliest spice of the world. Over the past few years, considerable interest has developed in saffron because of high pharmaceutical and industrial properties. In addition to non-volatile active components such as lycopene, carotenoids and zeaxanthin, *Crocus* contains several aromatic and volatile compounds [1]. The colour, bitter taste, and aroma are its three main and peculiar characteristics, which are conferred by apocarotenoids: crocin, picrocrocin, and safranal, respectively [2]. The cultivated saffron species (*C. sativus*), belonging to family Iridaceae, is a sterile triploid mutant of *C. cartwrightianus* (native of east Mediterranean area) [3]. Due to irregular meiosis there are several abnormalities during gametophyte development and sporogenesis thus producing abnormal pollen, whereas ovules of saffron remain viable which proves that infertility in this plant is mainly due to male gametophyte [4]. Due to the male sterility of saffron there are no chances of genetic improvement through sexual means. It is propagated vegetatively only, using corms, which is a major constraint in breeding better planting material and enhancing the quantity of saffron. A large corm above 8 gm produces three to four small daughter corms, which take 2 to 3 seasons for achieving the size and weight for flowering. The multiplication rate is very slow and the production further declines due to Fusarium corm rot, rodent infection, and other diseases which cause commercial loss to a great extent [5]. Due to inefficient clonal propagation in corms, large-scale propagation by tissue culture methods seems to be key for efficacious saffron plantlet development [6].

A parallel yet constructive and powerful technology which has been extensively used for crop improvement is the production of transgenics. Although, the application of these technologies has been hampered by the apprehension of potential off-target mutagenesis brought about by the expression or presence of transgenes, CRISPR/Cas9 mediated genome editing has been promoted as an attractive alternative for its precise editing strategy. It is the newly discovered gene editing tool that allows changing any DNA sequence in a precise manner [7]. Recently, the technique has been used in some plants [8, 9]; however, there are no reports so far in saffron. This new emerging technology guarantees to alter the progress of biotechnological research by modulating target genes involved in plant metabolism, immunity and stress tolerance to generate crops with desired improvements [10]. The off-site targeting is minimised to a greater extent with the advent of various in silico tools which provide a means of selecting the guide RNA of choice [11].

In view of the fact that Saffron is an economically and medicinally important plant, several efforts for genetic improvement through biotechnological approaches have been made in the past, however, limited success has been achieved. In order to extend CRISPR-cas mediated genome editing to Saffron, development of an efficient transformation system is a pre-requisite. Thus, with the aim of developing a platform for stable transformation in saffron, first a promising in vitro protocol was developed. The experiments were laid for efficient production of callus and somatic embryos. Further differentiation of these tissues led to high frequency of cormlet production. Once the protocols were established, strategies for achieving stable transformation system was developed using Cas9 gene. These leads will prove highly useful for CRISPR-cas9 mediated gene editing in Saffron for enhancing the apocarotenoid content.

## Results and discussion

### Callus production from corm slices in vitro and regeneration

Direct and indirect organogenesis has been significantly achieved for efficient regeneration of saffron using a variety of explants. Indirect organogenesis refers to the differentiation of various organs like shoots and/or roots from the callus. Direct organogenesis does not involve callus intermediate and therefore, the various organs are developed directly from the tissue [12]. Indirect organogenesis is efficient in producing transgenic plants, whereas for micropropagation purpose, direct organogenesis is a more coherent technique. Direct and indirect organogenesis was reported by Zeybek et al. in 2012 using MS medium supplemented with various plant growth regulators [13]. Subsequently, another group reported cormlet production via somatic embryogenesis, using MS medium containing TDZ and picloram [14].

Many researchers have favoured the use of leaf and shoot explants due to ease of availability. Efforts for initiating in vitro regeneration by involving the reproductive parts of the plant such as ovary, stigma, style and whole bud have also been made by several researchers [15]. Our studies involved the use of corm slices as explants since corms are available almost throughout the year, have large reserve of dividing meristematic cells and cells associated with vascular tissues, that have the potential for giving rise to organ primordia [16].

Saffron corm as the source of explant, presents a major challenge of contamination, since corms are grown under the soil, and have bacterial and fungal contaminations. Therefore, corms have to be properly sterilized. For mitigating infections, three-step sterilization has been proposed [17] that achieved aseptic cultures with ~ 81% explant survival. The process involved the blended use of 0.1% carbendizime, 0.2% mancozeb, 50% sodium hypochlorite and 1.6% mercuric chloride. Although, use of each of these sterilants singly led to effective asepsis, yet the survival got badly affected, while combinatorial treatment increased explant asepsis with higher survival percentage [17]. In our study, two-step surface sterilization process was optimized involving the individual use of 0.1% mercuric chloride and 4% sodium hypochlorite and resulted in efficient asepsis with 86% of explant survival. We achieved efficient disinfection of the corms by not involving strong chemicals such as carbendizime, mancozeb and/or mercuric chloride that have a property of corrosive sublimates in addition to their effective surface sterilization property.

During the last two decades or so, number of media have been used by various researchers for in vitro studies in Saffron. Comparative studies have been undertaken between Murashige and Skoog (MS) and Linsmaier and Skoog (LS) media [18], LS for callogenesis and organogenesis [19], B5 for indirect and direct regeneration [20] and 1/2MS for direct regeneration of shoot in saffron [21]. In the present studies we used complete MS medium as it has been shown to be the best suited medium for complete propagation of saffron.

The imperative function rendered by PGRs (Plant Growth Regulators) in various stages of plant micropropagation is reflected by the scores of supplemented media formulations used for saffron, largely dependent on the explant used [22]. Overall, in direct organogenesis experiments, various combinations of PGRs such as zeatin, thidiazuron (TDZ), alpha-naphthalene acetic acid (NAA), 6-Benzylaminopurine (BAP), N^6^-furfurylaminopurine (Kn), indole-3-acetic acid (IAA), 2,4-dichlorophenoxy acetic acid (2,4-D) have been used. Furthermore, in general, PGRs used by earlier researchers were a combination of auxins and cytokinins, similar to the combinations in the present investigation (BAP+NAA, TDZ+NAA, BAP+IAA, 2,4-D+BAP+IAA, 2,4-D+IAA and 2,4-D+BAP).

In the present case, the corm slices started swelling in 2 weeks and began to turn pale yellow in colour due to the release of certain phenolic compounds. After 3 to 4 weeks, pale yellow coloured unorganized mass of cells started appearing that later resulted into well organized, white friable callus, in the medium containing 2,4-D (0.5 mg/L), BAP (1 mg/L) and IAA (1 mg/L), with more than 70% multiplication rate (Table 1). In another set of experiments MS medium + 2,4-D (1 mg/L) + IAA (1 mg/L) also showed induction of callus, however, with a very less rate of multiplication ~ 30% (Table 1). Only those media have been listed on which more than two calli were induced after 4 weeks.

**Table 1:** Effect of plant growth regulators in combination with MS medium, on callus induction from corms of saffron

Combination of Plant growth regulators used					Response
BAP (mg/L)	NAA (mg/L)	2,4-D (mg/L)	TDZ (mg/L)	IAA (mg/L)	Number of explants forming calli/petriplate (n = 5) Mean ± SE
1.0	0.5	–	–	–	3.6 ± 0.00049
–	4.0	–	4.0	–	5.0 ± 0.0081
1.0	–	–	–	–	4.0 ± 0.0081
1.0	–	0.5	–	1.0	7.6 ± 0.0049
	–	1.0	–	1.0	3.8 ± 0.011
6.0	–	1.9	–	–	4.6 ± 0.049

Number of explants inoculated per petriplate was ten; five replicates of each combination were laid (total number of explants 50/combination). Data was collected after 8 weeks. Sucrose concentration in all the media was 3 g/L

**Fig. 1:**
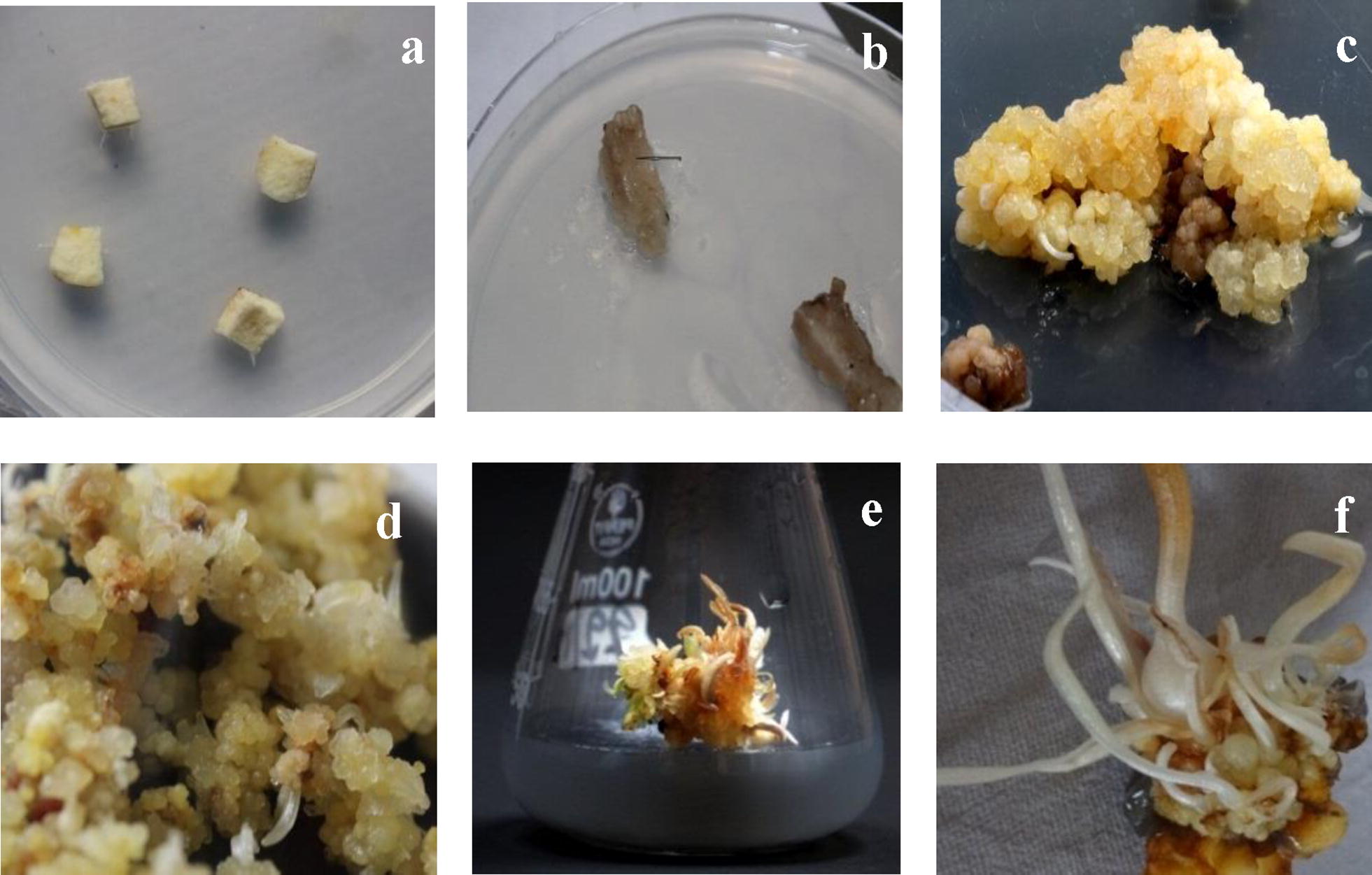
In vitro development of microcorms as a means of cormlet regeneration. The inserts include **a** corms as explant on first day of inoculation **b** callus induction on complete MS media supplemented with 2,4-D (0.5 mg/L), BAP (1 mg/L), IAA (1 mg/L) after 2 weeks of culture (**c**) raised embryogenic calli using complete MS medium supplemented with TDZ (0.5 mg/L) + IAA (1 mg/L) + Activated charcoal (0.1 g/L) after 4 weeks of culture (**d**) multiplied somatic embryos maintained on using complete MS medium supplemented with TDZ (0.5 mg/L) + IAA (1 mg/L) + Activated charcoal (0.1 g/L) after 2 weeks of culture (**e**) *In vitro* shoot initiation after 4 weeks of culture (**f**) developed cormlets formed from proliferated calli after 8 weeks of culture

Temperature and illumination have been observed to considerably influence callus induction and regeneration, besides regulating the accumulation of secondary metabolites [23]. Generally, for in vitro organogenesis, a temperature range of 17–25 °C has been demonstrated to influence the induction and proliferation of the callus. It has been established that both temperature and light influence growth of explants in a varied but inclusive manner. Callus has been obtained under dark conditions at 20–25 °C [24], 16 h light at 25 °C [25], 16 h light 3000 lx intensity, incubated at 15 ± 1 °C [26], 1500 lx at 25 ± 3 °C, shoot generation at 20 °C and rooting at 15 °C with 14 h photoperiod [27]. The present study was undertaken at standard conditions of photoperiod 16/8 h, 45% relative humidity at 20 ± 2 °C (Fig. 1) and resulted in successful generation of callus followed by organogenesis.

### Cytokinins, auxins and carbohydrate source for cormlet production

During somatic embryogenesis the pace of cell division and protein synthesis is rapid, thus higher supply of purine and purine metabolites is required. TDZ enhances the supply of purines and is therefore, considered important for the somatic embryogenesis in plants. Chemical structural studies confirm TDZ to be highly effective due to its diphenylurea derivative yielding 50–100 times more influential cytokinin activity [24]. Among the cytokinins used in earlier studies, TDZ was independently capable to induce shoot regeneration whereas BAP, zeatin and Kn required auxins to stimulate multiple shoots [20]. Parray et al. demonstrated very high efficiency of TDZ in regenerating over 70 cormlets per slice of corm when they used 1/2MS supplemented with TDZ and IAA [21]. In our study, significant increase in biomass and mean number of somatic embryos was observed after using TDZ with IAA in the medium (Table 2).

**Table 2:** Effect of plant growth regulators in combination with MS medium on somatic embryogenesis

Combinations of Plant growth regulators used							Response
BAP (mg/L)	NAA (mg/L)	TDZ (mg/L)	IAA (mg/L)	KIN (mg/L)	Picloram (mg/L)	Activated Charcoal (mg/L)	Number of somatic embryos formed/flask (n = 5) Mean ± SE
–	–	8.8	3.0	1.0	–	–	2.6 ± 0.0048
2.0	0.5	–	–	–	–	–	5.6 ± 0.0048
–	–	1.0	2.6	–	–	–	4.0 ± 0.008
–	–	0.5	–	–	0.5	–	4.4 ± 0.004
–	–	1.0	0.5	–	–	–	5.8 ± 0.012
–	–	0.5	1.0	–	–	0.1	8.4 ± 0.004

Number of explants inoculated per flask was ten; five replicates of each combination were laid (total number of explants 50/combination). Data was collected after 8 weeks. Sucrose concentration in all the media was 3 g/L

Studies undertaken for over last four decades have confirmed effective regeneration of callus and intact plantlets from corm explants in media fortified with IAA and/or 2,4-D with the addition of cytokinins [27]. Some of the interesting observations include; induction of embryonic callus using 2,4-D+Kn [28], non-embryonic callus with NAA+BAP [29], multiple shoot generation using NAA+BAP [30] and development of salt tolerant saffron using 2,4-D+BAP [31]. Out of the different combinations of plant growth hormones used in this study, MS medium supplemented with TDZ 0.5 mg/L+IAA 1 mg/L+ activated charcoal 0.1 g/L at 15 ± 2 °C resulted into white, shining, filled somatic embryos in 8 weeks (Table 2), whereas the other concentrations of TDZ and IAA resulted into pale yellow, hard non-embryogenic structure. The added feature of activated charcoal at 0.1 g/L had a positive effect on growth rate of somatic embryos by reducing tissue and medium browning, phenolic exudations (which hinder certain growth stages in plants) and peroxidase activity. Many other researchers have used activated charcoal in addition to the PGRs to improve the frequency of regeneration in saffron [21, 32–34].

A large energy reserve required by the shoots for conversion into a cormlet is provided by a good amount of carbon source. A pioneering study undertaken by Ding et al. established micropropagation of saffron by supplementation of MS medium with 3% sucrose [35]. Recent studies, however, have established direct organogenesis by the addition of sucrose up to the levels of 8% [36] and 9% [37]. Devi et al. established that sucrose levels at 6% aided the formation of nodular and hard structures with inhibited proliferation [25]. In our study, high concentration of sucrose was integrated with the combination of PGRs for gaining the efficiency over a period of 90 days. The combination of MS medium with TDZ, IAA, activated charcoal and 4% sucrose showed the best results with 68% efficiency (Table 3). Lower concentration of sucrose resulted in slow growth whereas concentration higher than 4% resulted in cellular death indicated by the blackening of the callus.

**Table 3:** Effect of plant growth regulators in combination with MS medium on cormlet formation from in vitro raised shoots

Combination of PGRs used with other supplements								Response
BAP (mg/L)	NAA (mg/L)	TDZ (mg/L)	IAA (mg/L)	IBA (mg/L)	Sucrose (g/L)	Activated charcoal (g/L)	Paclabutrazole (mg/L)	No. of cormlets formed/flask (n = 5) Mean ± SE
4.5	8.0	–	–	–	3.0	–	–	1.6 ± 0.0048
–	–	1.0	–	0.5	3.5	–	2.0	0.2 ± 0.0032
–	–	0.5	1.0	–	4.0	0.1	–	6.8 ± 0.004
–	–	3	2	–	3.0	–	–	2.0 ± 0.008
–	–	1	0.5	–	6.0	–	–	2.2 ± 0.004
–	–	1	0.5	–	8.0	–	–	1.2 ± 0.004

Number of explants inoculated per flask was ten; five replicates of each combination were laid (total number of explants 50/combination). Data was collected after 8 weeks

### *Agrobacterium tumefaciens* mediated genetic transformation in Saffron

The use of *Agrobacterium*-mediated genetic transformation has been favoured mainly due to stable integration of a single copy of the gene of interest, with minimum or no rearrangements of the foreign DNA structure, resulting in few complications such as gene co-suppression, instability or silencing [38]. *A. tumefaciens* has natural ability to alter plant genetic makeup and has been used for genetic transformation in several plants. Stable transformation using this technique has not been achieved in saffron.

Studies that have favoured the use of *Agrobacterium* in monocots have propounded that the success or failure of transformation largely depends on the co-cultivation period. This period overlaps with the S-phase of the cell-cycle that is essential for T-DNA transfer from *Agrobacterium* to the geophyte. Therefore, pre-determination of this period helps overcome less frequency of transformation or overgrowth of *Agrobacterium*. Previous studies of geophytes have established a co-cultivation period of 3 days as best for *Agapanthus* species [39] whereas a 3-day period resulted in highest GUS expression levels in *Typha latifolia* [40]. Interestingly, our study demonstrated 2-day period of co-cultivation as optimum for Cas9 expression in saffron.

**Fig. 2:**
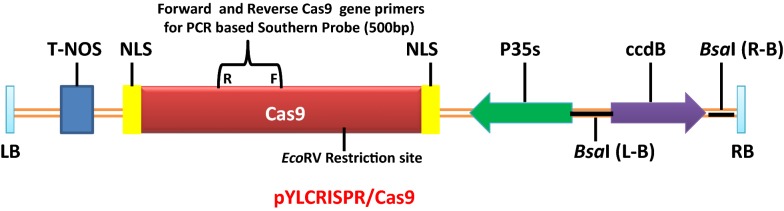
Schematic representation of the T-DNA region of the binary vector pYLCRISPR/Cas9 (modified pCAMBIA1300 backbone) developed in-house and utilized for *Crocus sativus* agro-mediated transformation revealing the 35S promoter:Cas9:NOS terminator cassette; MCS for single or multiple sgRNA insertion; RB: right border; LB: left border

**Fig. 3:**
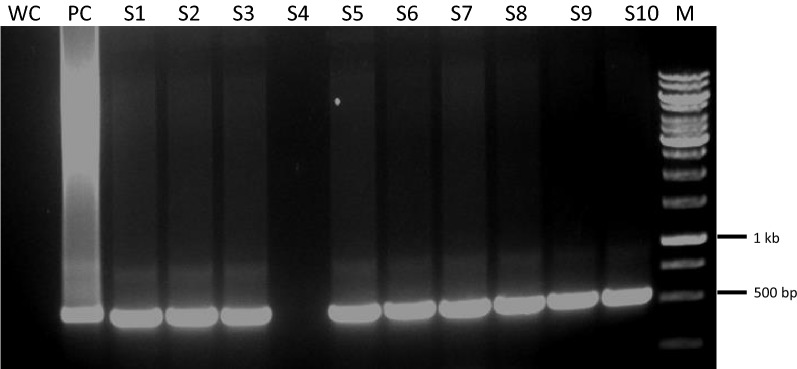
PCR analysis to screen putative transgenic T_0_*Crocus sativus* lines. Cas9 plasmid was used as positive control (P); WC represents water control; Lanes 3–12 represent the putative T_0_ transgenic lines of *Crocus sativus* expressing Cas9 gene; Lane 13 (M) represents Molecular Ladder

**Fig. 4:**
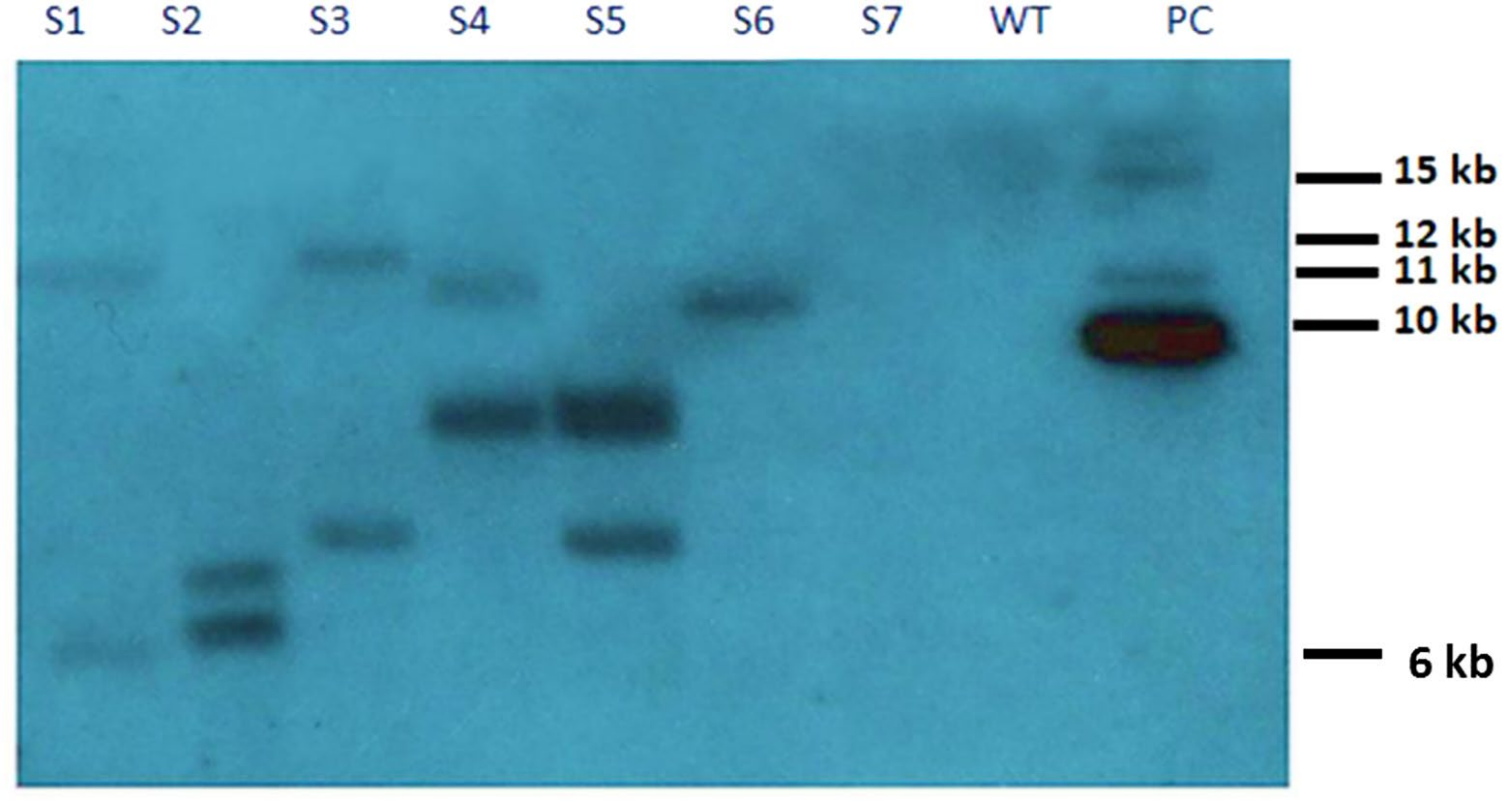
Southern blot showing the differential DNA copy number of Cas9 gene in various transgenic T_0_*Crocus sativus* lines. The figure represents: PC as positive control; WT as wild type and lanes 1–7 demonstrate different transgenic saffron lines. These observations were obtained by the digestion of genomic DNA from seven transgenic plants using restriction enzyme EcoRV and Cutsmart 2 buffer obtained from NEB

In the present study a binary vector was constructed to improve the delivery of Cas9 gene in a single expression vector for *Agrobacterium* and the plant, under a 35S promoter (Fig. 2). Additionally, no selectable marker genes were included to identify transgene in plants as the present effort was made to overcome the recalcitrance of saffron towards genetic transformation. Positive clones of *Agrobacterium tumefaciens* (EHA105) carrying the Cas9 gene were detected on YEPD-Kanamycin resistant medium. Further detection was confirmed by polymerase chain reaction using CRISPR-Cas9 specific primers as mentioned in the materials and methods section (Fig. 3). Subsequently, molecular detection of specific exogenous DNA (Cas9 copy number) present in the transformed plants was analysed by Southern blot analysis (Fig. 4). The genomic DNA isolated from seven transgenic plants regenerated from different calli, was digested with EcoRV. There is only one EcoRV restriction site in the T-DNA Cas9 region. After hybridization with Cas9 gene probe (500 bp) followed by autoradiography, hybridization signals corresponding to fragments of different sizes ranging from 6 to 12 kb, were observed (Fig. 4) in different transgenic plants. The pattern of hybridization confirms the presence of our target exogenous DNA in several copies and at random locations in the transformed saffron. The detected bands clearly demonstrate the successful integration of T-DNA into Saffron genome and also robustness of the technique to detect high molecular weight bands. No such signal was observed in the untransformed control plant (Fig. 4). The best transformation efficiency of 4% was achieved with 35 days old somatic embryos cultured in presence of cefotaxime (Table 4).

**Table 4:** Transformation efficiency relative to the experiment in which resistant callus or transgenic plants were produced

Type of explant	Age of the explant (in days)	Inoculated embryo (A)	No. of calli produced in media with cefotaxime	Transgenic plants (B)	Transformation efficiency (B/A %)
Immature embryo	30	52	2	0	0
Immature embryo	40	50	3	1	2
Immature embryo	35	50	4	0	0
Somatic embryo	30	120	8	3	3
Somatic embryo	35	56	5	2	4
Somatic embryo	40	98	6	3	3

## Conclusion

We have developed a method to circumvent the limited regeneration potential of saffron to produce transgenics, in addition to standardizing the protocol for callus initiation and cormlet production. The standardised protocol for cormlet generation had a transformation/regeneration efficiency of 4% that can be used for further propagation of *Crocus sativus*. This pilot study generated stable transgenic *Crocus* calli where Cas9 has been integrated in its genome. This work certainly validates the viability of this crop for gene editing studies.

## Materials and methods

### Explant and sterilization

For the present investigation the corms were collected from the fields at Pampore, Jammu and Kashmir, India located at 34.02° N/74.93° E at an elevation of 1573 m (5161 ft). Corms were thoroughly washed under tap water followed by tunic removal, dipped in Tween-20 for 5 min and then transferred to sterile environment for aseptic sterilization. Surface sterilization was performed by soaking the corms in 0.1% mercuric chloride for 2 min followed by 4% sodium hypochlorite for 5 min. Finally the corms were washed with autoclaved distilled water 5 times to remove residual chemicals. Aseptic corms were sliced into 0.5–1 cm^3^ corm parts including apical or lateral meristematic nodes for callus induction and inoculated on solidified Murashige and Skoog (MS) medium plates (pH 5.8 ± 0.2). For optimizing best suited medium for callus induction MS medium supplemented with sucrose (3%), 0.8% agar and different plant growth regulators at various concentrations were used. The autoclavable plant growth regulators like BAP, IBA, and NAA were added before autoclaving while heat sensitive plant growth regulators like zeatin, TDZ were filter sterilized and added after autoclaving. Cultures were kept under standard culture conditions of 16/8 h, 45% relative humidity at 20 ± 2 °C.

### Selection of various plant growth regulators (PGRs)

One of the critical factors involved in the successful genetic transformation is the development of a high efficiency in vitro regeneration protocol. Since good number of publications were available on in vitro studies in saffron, therefore, selection of various media with specific composition was made on the basis of critical examination of these reports. A systematic approach was used for selection of PGRs. Further, concentrations and combinations were modified so as to improve the frequency of cormlet regeneration. In order to arrive at suitable media resulting in highly efficient response, all the combinations with varying concentrations were used. However, only the ones showing positive response were pursued further (Tables 1 and 2). The rationale for inclusion of BAP, NAA, TDZ, IBA, IAA, KIN, Picloram, Activated charcoal, is based on the results obtained by various workers who have published the protocols from time to time [15, 41–43].

### Callus initiation and cormlet production

Sterilized corm slices were inoculated on different culture media such as: (a) MS + BAP (1 mg/L) + NAA (0.5 mg/L) (b) MS + TDZ (4 mg/L) + NAA (4 mg/L) (c) MS + BAP(1 mg/L) + IAA (0.5 mg/L) (d) MS + 2,4-D(0.5 mg/L) + BAP (1 mg/L) + IAA (1 mg/L) (e) MS + 2,4-D (1 mg/L) + IAA (1 mg/L) (f) MS + 2,4-D (1.9 mg/L) + BAP (6 mg/L). Ten explants were selected and inoculated in five replicates of each combination for optimizing the best combination. Calli were further sub-cultured on media supplemented with different concentrations of plant growth regulators (PGRs) for multiplication and optimization of somatic embryos: (a) MS + KIN (1 mg/L) + TDZ (8.8 mg/L) + IAA (3 mg/L) (b) MS + BAP (2 mg/L) + NAA (0.5 mg/L) (c) MS + TDZ (1 mg/L) + IAA (2.6 mg/L) (d) MS + picloram (0.5 mg/L) + TDZ (0.5 mg/L) (e) MS + TDZ (1 mg/L) + IAA (0.5 mg/L) (f) MS +TDZ (0.5 mg/L) + IAA (1 mg/L) + activated charcoal (0.1 g/L). Embryos were then sub-cultured on media containing different concentrations of BAP (1 mg/L to 10 mg/L) and activated charcoal (0.1 to 0.5 mg/L) for shoot induction. Further, the shoots were sub-cultured on media having sucrose as carbon source, along with different combination of PGRs to standardize cormlet production such as: (a) MS + BAP (4.5 mg/L) + NAA (8 mg/L) (b) MS + TDZ (1 mg/L) + IBA (0.5 mg/L) + paclabutrazol (2 mg/L) (c) MS + TDZ (0.5 mg/L) + IBA (1 mg/L) + sucrose (40 g/L) + Activated charcoal (AC) 0.1 g/L (d) MS + TDZ (1 mg/L) + IAA (0.5 mg/L) + sucrose (30 g/L) (e) MS + TDZ (1 mg/L) + IBA (0.5 mg/L) + sucrose (60 g/L) (f) MS + TDZ (1 mg/L) + IBA (0.5 mg/L) + sucrose (80 g/L).

### Design of CRISPR/Cas9 vector

Saffron codon optimized Cas9 (SpCas9) gene sequence with attached nuclear localization signals (NLSs) at both ends were designed, including rich GC content at the 5′ terminal region by following Ma et al. [44]. Cas9 sequence was linked to the promoter in intermediate plasmids, and the cassette was cloned into binary vector pYLCRISPR/Cas9P_35S_(Addgene). The pYLCRISPR/Cas9 was derived from pCAMBIA1300 and introduced into a BsaI restriction enzyme recognition site in the multiple cloning sites. Two sites having BsaI can be used for the assembly of single or multiple sgRNAs (Fig. 2).

### Agrobacterium-mediated transformation

*Agrobacterium tumefaciens* strain EHA 105 and the binary vector (pYLCRISPR/Cas9) containing the Cas9 gene linked to the 35S promoter were used. A binary vector, pYLCRISPR/Cas9 was constructed with the cDNA of the Cas9 gene. Plasmid pYLCRISPR/Cas9 was introduced into *A. tumefaciens* (EHA105) strains by freeze–thaw method. *Agrobacterium* cultures were plated on Luria–Bertani (LB) medium supplemented with 50 mg/L kanamycin and grown for 3 days at 28 °C to form colonies. Each single colony with a diameter of 1 mm was picked up and cultured in 20 mL LB liquid containing the same antibiotic. The culture was agitated at 120 rpm for 20 h at 28 °C. After adjusting the optical density to 0.6 units at 600 nm (OD600 nm), the *Agrobacterium* cultures were used for transformation experiment.

Calli that were mildly injured using a scalpel were precultured for 3 days prior to *Agrobacterium* infection and were immersed in *Agrobacterium* suspension for 30 min with an optimized acetosyringone concentration of 100 µM. The explants were then blotted dry on sterile filter paper and co-cultivated for 3 days in the dark at 22 °C on hormone-free MS medium. After the 3 day co-cultivation period, the buds were transferred to fresh medium of the same composition but without acetosyringone, together with 200 mg/L cefotaxime for 5 days and transferred back to MS liquid medium with the antibiotic of the same concentration, for another 5 days and then transferred to MS solid medium. It may be worthwhile to mention here that cefotaxime is not a selectable marker and is an antibiotic commonly used for the treatment of plant tissue infections, caused by the gram-negative bacteria. As a result, the tissue steers clear of contamination during *Agrobacterium tumefaciens* mediated plant transformation. Cefotaxime has very low toxicity in plants (concentrations up to 500 mg/L). In the present case, we used cefotaxime in callus induction medium to get resistant calli i.e. the calli without bacterial contamination after *Agrobacterium mediated transformation.* After 2 weeks they were transferred again to fresh liquid medium in conical flasks and kept for 5 days. During this period, dead buds were removed before being sub-cultured back to MS medium. Single buds were separated from multiple bud clumps and sub-cultured onto solid MS medium with TDZ 0.5 mg/L + IAA 1 mg/L + Activated charcoal 0.1 g/L until the single plants were regenerated. All plants regenerated from each putatively independent transformed bud line were maintained under in vitro conditions.

### PCR analysis of T_0_ Cas9 *Crocus sativus* transgenic lines

Genomic DNA from the different T_0_ putative Cas9 lines of *Crocus sativus* was extracted using the Cetyl Trimethyl Ammonium Bromide (CTAB) method and further used for polymerase chain reaction amplification. The PCR mix contained 1.5 mM MgCl_2_, 150 ng of each Cas9 gene primers (F-5′-CGACTTACCCTTCTCCACCTT-3′; R-5′-ACGCTCACGATGCTTACCTT-3′), 10 mM dNTPs, 2.5U Taq DNA polymerase and 200 ng genomic DNA. The reaction mixture was set to 50 μL and placed in a thermal cycler (Bio-Rad Laboratories, USA) using an initial denaturing time of 3 min at 94 °C, followed by 30 cycles of denaturation for 1 min at 94 °C, annealing at 55 °C for 1 min, extension for 1 min at 72 °C and final extension for 6 min at 72 °C. A 3 μL product from the first reaction was used as template in the second reaction that involved nested pair primers with PCR conditions similar to the external primer run. The amplified products were separated and visualized in 0.8% agarose gels stained with 1 μg/mL ethidium bromide.

### Southern blot analysis

Genomic DNAs (25 μg) from the WT, putative T_0_ transgenic lines and positive control plasmid (1 ng) were digested with EcoRV enzyme in a 40 µL reaction, overnight. The digestion products were separated by electrophoresis in a 0.8% (w/v) agarose Tris borate ethylenediaminetetraacetic acid (TBE) gel at 25 V, 15 mA overnight. The gel was depurinated using 0.25 M HCl for 10 min followed by washing in deionized water on a slow rotating shaker for 5 min, twice (15 min each) in denaturation buffer solution (0.5 M NaOH, 1.5 M NaCl) and then neutralized two times in a neutralization buffer (1 M Tris, 1.5 M NaCl, pH 7) for 30 min each. The gel was soaked in 20× standard sodium citrate (SSC) for 5 min. The DNA was transferred onto a positively charged nylon membrane (Millipore, India; cat. no. 7104633) by capillary blotting under 20× SSC conditions (pH 7) [45]. The blot was washed in 2× SSC for 5 min and DNA was fixed onto the blot by UV cross-link for 45 s with a UV transilluminator. The blot was wet on both sides using 5× SSC and pre-hybridized for one hr using DIG EasyHyb buffer solution (Roche, Germany). The blot was then hybridized at a temperature of 68 °C overnight using DIG EasyHyb buffer solution (Roche, Germany) in which 1 µL of the denatured DIG labelled Cas9 probe generated using PCR DIG probe synthesis kit was included. The blot was washed twice in stringent low washing buffer solution (2× SSC, 0.1% (w/v) SDS at room temperature for 5 min, followed by washing using 0.5× SSC plus 0.1% (w/v) SDS and then 0.1 × SSC plus 0.1% (w/v) SDS buffer solution at a temperature of 68 °C each lasting 10 min. Chemiluminescent detection was performed as described in the users’ instruction manual (Roche Diagnostics, Germany). The signals were visualized using X-ray detection film.

## Data Availability

All data generated or analysed during this study are included in this published article. The data obtained from in vitro regeneration experimentation was analysed using analysis of variance (ANOVA) of completely randomized design. The groups that showed variance were then subjected to Duncan’s Multiple Range Test 10 with a significance value at P > 0.05.

## Abbreviations

CRISPR: Clustered Regularly Interspaced Short Palindromic Repeats
PCR: Polymerase chain reaction
DNA: Deoxyribonucleic acid
Cas: Caspases
LS: Linsmaier and Skoog
PGR: Plant growth regulators
mg: Milligrams
L: Litres
T-DNA: Transfer DNA
GUS: β-Glucuronidase
YEPD: Yeast extract peptone dextrose
DIG: Digoxigenin
OD: Optical density
HCl: Hydrochloric acid
SDS: Sodium dodecyl sulphate

## References

[1] Bukhari SI, Manzoor M, Dhar MK (2018). A comprehensive review of the pharmacological potential of *Crocus sativus* and its bioactive apocarotenoids. Biomed Pharmacother.

[2] Dar RA, Shahnawaz M, Malik SB, Sangale MK, Ade AB, Qazi PH (2017). Cultivation, distribution, taxonomy, chemical composition and medical importance of *Crocus sativus*. J Phytopharmacol..

[3] Kashtwari M, Wani AA, Dhar MK, Jan S, Kamili AN (2018). Development of an efficient in vitro mutagenesis protocol for genetic improvement of saffron (*Crocus sativus* L.). Physiol Mol Biol Plants..

[4] Dhar MK, Sharma M, Bhat A, Chrungoo NK, Kaul S (2017). Functional genomics of apocarotenoids in saffron: insights from chemistry, molecular biology and therapeutic applications. Brief Funct Genomics..

[5] Sharma M, Kaul S, Dhar MK (2019). Transcript profiling of carotenoid/apocarotenoid biosynthesis genes during corm development of saffron (*Crocus sativus* L.). Protoplasma..

[6] Nehvi FA, Yasmin S. Advance in saffron research for integrated development of saffron in Kashmir, India. In: Vth International Symposium on Saffron Biology and Technology: Advances in Biology, Technologies, Uses and Market. 2016 Nov 23; 1184 (p. 63–68).

[7] Chen K, Wang Y, Zhang R, Zhang H, Gao C (2019). CRISPR/Cas genome editing and precision plant breeding in agriculture. Annu Rev Plant Biol.

[8] Zhang Q, Zhang Y, Lu MH, Chai YP, Jiang YY, Zhou Y, Wang XC, Chen QJ (2019). A novel ternary vector system united with morphogenic genes enhances CRISPR/Cas delivery in maize. Plant Physiol.

[9] Shimatani Z, Kashojiya S, Takayama M, Terada R, Arazoe T, Ishii H, Teramura H, Yamamoto T, Komatsu H, Miura K, Ezura H (2017). Targeted base editing in rice and tomato using a CRISPR-Cas9 cytidine deaminase fusion. Nat Biotechnol.

[10] Li R, Zhang L, Wang L, Chen L, Zhao R, Sheng J, Shen L (2018). Reduction of tomato-plant chilling tolerance by CRISPR–Cas9-mediated SlCBF1 mutagenesis. J Agric Food Chem..

[11] Wolt JD, Wang K, Sashital D, Lawrence-Dill CJ (2016). Achieving plant CRISPR targeting that limits off-target effects. Plant Genome..

[12] Bhatia S, Bera T, Bhatia S, Sharma K, Dahiya R, Bera T (2015). Somatic embryogenesis and organogenesis. Modern applications of plant biotechnology in pharmaceutical sciences.

[13] Zeybek E, Önde S, Kaya Z (2012). Improved in vitro micropropagation method with adventitious corms and roots for endangered saffron. Cent Eur J Biol.

[14] Devi K, Sharma M, Singh M, Singh Ahuja P (2011). In vitro cormlet production and growth evaluation under greenhouse conditions in saffron (*Crocus sativus* L.)–a commercially important crop. Eng Life Sci.

[15] Ahmad M, Razvi SM, Zaffar G, Nengroo ZI, Mir SD, Wani BA, Ameeque A, Habib M (2014). Micropropagation of saffron (*Crocus sativus* L.): a review. J Cell Tissue Res.

[16] Ahrazem O, Rubio-Moraga A, Nebauer SG, Molina RV, Gomez-Gomez L (2015). Saffron: its phytochemistry, developmental processes, and biotechnological prospects. J Agric Food Chem..

[17] Yasmin S, Nehvi F (2014). In vitro microcorm formation in saffron (*Crocus sativus* L.). J Cell Tissue Res..

[18] MalekZadeh S, Khosrowshahli M, Taeb M (2009). Cryopreservation of the axial meristem of *Crocus sativus* L.. Cryobiology.

[19] Mir JI, Ahmed N, Wani SH, Rashid R, Mir H, Sheikh MA (2010). In vitro development of microcorms and stigma like structures in saffron (*Crocus sativus* L.). PhysiolMol Biol Plants..

[20] Sharifi G, Ebrahimzadeh H, Ghareyazie B, Karimi M (2010). Globular embryo-like structures and highly efficient thidiazuron-induced multiple shoot formation in saffron (*Crocus sativus* L.). Vitro Cell Develop Biol Plant..

[21] Parray JA, Kamili AN, Hamid R, Husaini AM (2012). In vitro cormlet production of saffron (*Crocus sativus* L. *Kashmirianus*) and their flowering response under greenhouse. GM Crops Food..

[22] Vahedi M, Kalantari S, Salami SA (2014). Factors affecting callus induction and organogenesis in saffron (*Crocus sativus* L.). Plant Tissue Cult Biotechnol..

[23] Verma SK, Das AK, Cingoz GS, Uslu E, Gurel E (2016). Influence of nutrient media on callus induction, somatic embryogenesis and plant regeneration in selected Turkish crocus species. Biotechnol Rep..

[24] Vatankhah E, Niknam V, Ebrahimzadeh H (2014). Histological and biochemical parameters of *Crocus sativus* during in vitro root and shoot organogenesis. Biol Plant.

[25] Devi K, Sharma M, Ahuja PS (2014). Direct somatic embryogenesis with high frequency plantlet regeneration and successive cormlet production in saffron (*Crocus sativus* L.). South Afr J Botany..

[26] Plessner O, Ziv M, Negbi M (1990). In vitro corm production in the saffron crocus (*Crocus sativus* L.). Plant Cell Tissue Organ Cult.

[27] Gantait S, Sinniah UR (2012). Rapid micropropagation of monopodial orchid hybrid (Aranda Wan Chark Kuan ‘Blue’ × Vanda coerulea Grifft. ex. Lindl.) through direct induction of protocorm-like bodies from leaf segments. Plant Growth Regul.

[28] Zaffar G, Wani SA, Anjum T, Zeerak NA. Colchicine induced variability in saffron. In: International Symposium on Saffron Biology and Biotechnology 2003, p. 277–280).

[29] Chaloushi B, Zarghami R, Abd-Mishani C, Omidi M, Agayev YM, Pakdaman Sardood B (2007). Effects of different hormonal treatments on the callus production and plantlet regeneration in saffron (*Crocus sativus* L.). Pak J Biol Sci.

[30] Darvishi E, Zarghami R, Mishani CA, Omidi M (2007). Effects of different hormone treatments on non-embryogenic and embryogenic callus induction and time-term enzyme treatments on number and viability of isolated protoplasts in saffron (*Crocus sativus* L.). Acta Hortic.

[31] Shahabzadeh Z, Heidari B, Dadkhodaie A (2013). Regenerating salt tolerant saffron (*Crocus sativus*) using tissue culture with increased pharmaceutical ingredients. J Crop Sci Biotechnol.

[32] Sharafi A, Mirmasoumi M, Moradi A, Azadi P, Gholami M, Bagheri K (2017). Thin cell layer, a suitable explant for in vitro regeneration of saffron (*Crocus sativus* L.). J Agric Sci Technol..

[33] Ahuja A, Koul S, Ram G, Kaul BL (1994). Somatic embryogenesis and regeneration of plantlets in satiron. Indian J Exp Biol.

[34] Akbarian MM, Sharifabad HH, Noormohammadi GH, Kojouri FD (2012). The effect of potassium, zinc and iron foliar application on the production of saffron (*Crocus sativus*). Ann Biol Res..

[35] Ding B, Bai SH, Wu Y, Wang BK (1979). Preliminary report on tissue culture of corms of *Crocus sativus*. Acta Botanica Sinica..

[36] Sharma KD, Rathour R, Sharma R, Goel S, Sharma TR, Singh BM (2008). In vitro cormlet development in *Crocus sativus*. Biol Plant.

[37] Zaffar G, Ahmad M, Shahida I, Razvi SM, Habib M, Ahmad A (2014). Effect of paclobutrazol and sucrose on in vitro corm formation in saffron (*Crocus sativus*). J Cell Tissue Res.

[38] Sood P, Bhattacharya A, Sood A (2011). Problems and possibilities of monocot transformation. Biol Plant.

[39] Suzuki S, Supaibulwatana K, Mii M, Nakano M (2001). Production of transgenic plants of the Liliaceous ornamental plant *Agapanthuspraecox* ssp. *orientalis* (Leighton) Leighton via Agrobacterium-mediated transformation of embryogenic calli. Plant Sci.

[40] Babu P, Chawla HS (2000). In vitro regeneration and *Agrobacterium* mediated transformation in gladiolus. J Hortic Sci Biotechnol.

[41] Kumar R, Singh V, Devi K, Sharma M, Singh MK, Ahuja PS (2008). State of art of saffron (*Crocus sativus* L.) agronomy: a comprehensive review. Food Rev Int.

[42] Sharma KD, Piqueras A (2010). Saffron (*Crocus sativus* L.) tissue culture: micropropagation and secondary metabolite production. Funct Plant Sci Biotechnol..

[43] Gantait S, Vahedi M (2015). In vitro regeneration of high value spice *Crocus sativus* L: a concise appraisal. J Appl Res Med Aromatic Plants..

[44] Ma X, Zhang Q, Zhu Q, Liu W, Chen Y, Qiu R, Wang B, Yang Z, Li H, Lin Y, Xie Y (2015). A robust CRISPR/Cas9 system for convenient, high-efficiency multiplex genome editing in monocot and dicot plants. Mol Plant..

[45] Song F, Zhang J, Gu A, Wu Y, Han L, He K, Chen Z, Yao J, Hu Y, Li G, Huang D (2003). Identification of cry1I-type genes from *Bacillus thuringiensis* strains and characterization of a novel cry1I-type gene. Appl Environ Microbiol.

